# A Review of Equine Laparoscopy

**DOI:** 10.5402/2012/492650

**Published:** 2012-10-24

**Authors:** Dean A. Hendrickson

**Affiliations:** ^1^American College of Veterinary Surgeons, USA; ^2^College of Veterinary Medicine and Biomedical Sciences, Colorado State University, Fort Collins, Colorado, USA

## Abstract

Minimally invasive surgery in the human was first identified in mid 900's. The procedure as is more commonly practiced now was first reported in 1912. There have been many advances and new techniques developed in the past 100 years. Equine laparoscopy, was first reported in the 1970's, and similarly has undergone much transformation in the last 40 years. It is now considered the standard of care in many surgical techniques such as cryptorchidectomy, ovariectomy, nephrosplenic space ablation, standing abdominal exploratory, and many other reproductive surgeries. This manuscript describes the history of minimally invasive surgery, and highlights many of the techniques that are currently performed in equine surgery. Special attention is given to instrumentation, ligating techniques, and the surgical principles of equine minimally invasive surgery.

## 1. History of Laparoscopy

Laparoscopy and thoracoscopy are endoscopic surgical techniques performed in the abdominal and thoracic cavities, respectively. A recent review by Spaner and Warnock describes the history of human endoscopy, laparoscopy, and laparoscopic surgery in detail [1]. In summary, we are the benefactors of all the work that has gone before us. The first reported use of reflected light to examine the cervix was by the Arabian physician Albukasim (936–1013 A.D.). The next reports were in the early 1800s where Bozzini used a mirror, illuminated by a wax candle, to examine the urethra. Many thought that this was simply a joy. In the mid-1800's Desormeaux developed a burner that used alcohol and turpentine on a WIC for illumination using open to endoscopy. Thermal injuries were the greatest concern to the structures being evaluated. George Kelling, in 1901, provided the 1st attempt at endoscopy of the peritoneal cavity. For insufflation he used sterile filtered oxygen and a cystoscope to look at the peritoneal cavity of liue dogs. Jakobaeus described 109 laparoscopys on 69 patients in 1912. The patients had conditions such as cirrhosis of the liver, metastatic cancer, and tuberculosis peritonitis. Later, Zollikofer used carbon dioxide to obtain pneumoperitoneum, which reduced pain and thermal complications. Kalk used a 135° forward viewing lens system and a dual trocar technique to simultaneously pass instruments into the abdomen. In 1935, Kalk wrote: “This method really does not deserve the widespread opposition that still exists today, normally based on total ignorance….” The first laparoscopic interventions were described in the 1930's. Fervers, performed adhesiolysis and diagnostic biopsies of abdominal organs. Cold light for classroom nation was developed in the early 1950s, reducing the possibilities of thermal burns during lab discomfort procedures. In the 1960s and 1970s, laparoscopy became an important part of gynecologic practice in people. The automatic insufflator was developed in 1977 by Semm, further improving the safety margin of laparoscopy. In the mid-1980s the video computer chip allowed laparoscopy to become integrated into general surgery.

Veterinary laparoscopy began much like the use of laparoscopy and people, in the field of gynecology. In 1970, Witherspoon and Talbot [2, 3] published 2 papers on the use of laparoscopy as a diagnostic tool to describe population events in the mare. Witherspoon and McQueen [4] also published on the development of an equine peritoneal fistula device which was in essence a cannula. Heinze et al., from Germany also published two papers in the 1970s on equine laparoscopy [5, 6]. In the 1980s, there were 4 papers published regarding equine laparoscopy. Witherspoon et al., in 1980, reviewed the current uses of laparoscopy or sous, and recommended the use of either a rigid or flexible endoscope and dual trocar techniques to allow surgical manipulation [7]. Wilson, in 1983 published on the use of laparoscopy to evaluate the reproductive tract of mares using a single trocar technique and a laparoscope alone for diagnostics, or an operating laparoscope for biopsies or manipulations [8]. Manipulations included: ovarian biopsy, biopsy of pelvic masses, bacterial culture of the infandibulum, aspiration of cysts, and examination for tubal patency. The 1st paper pushed on the use of laparoscopy and horses for something other than gynecological purposes was written by Fischer et al. in 1986 [9]. He and his coauthors performed laparoscopy in 5 clinically normal horses and 5 horses presented for abdominal abnormalities. They were successful in determining a diagnosis in all of the clinical cases. In 1989, Wilson and Madison [10] describe the use of laparoscopy to diagnose the presence and location of an abdominally retained testes. It wasn't until the 1990s that the use of laparoscopy for surgical intervention became commonplace. In one paper equine laparoscopy was compared to keyhole surgery in humans [11]. One literature search for equine laparoscopy revealed for manuscripts in the 1970s, 7 manuscripts in the 1980s, 27 manuscripts in the 1990s, and 62 manuscripts in the first decade of this century. It is obvious that equine laparoscopic surgery is here to stay and provides many benefits over traditional surgery for many surgical procedures [12].

Various surgical approaches and methods of hemostasis have been reported for intra-abdominal ligation and hemostasis in equine MIS. In recent years laparoscopic approaches have become popular in light of the fact that they are minimally invasive, provide superior visualization, allow tension free hemostasis, and when done in the standing position, eliminate the need for general anesthesia.

## 2. Challenges in Laparoscopic Surgery

 While there are many benefits to minimally invasive surgery, there are also some challenges. The main challenges are associated with performing surgery in a three-dimensional animal on a two-dimensional monitor, and the fulcrum effect of the body wall on instrument movement. It is difficult to perceived depth when working in a two-dimensional environment. One way to improve depth perception is to move the telescope closer to the area of interest when making fine movements. This means the camera operator needs to move the telescope as far away from the area of interest to insert other instruments, and move towards the area of interest at the same speed as the instruments, for fine motor movement. This requires good interaction between the surgeon and the assistant. The fulcrum effect of the body wall requires the surgeon and the assistant to recognize that the hand must move in the opposite direction of the desired movement of the tip the instrument for the telescope. This movement is compounded by the amount of instrument that has been introduced into the abdominal cavity. If most of the instrument has been introduced into the abdominal cavity the handle is moved slightly to achieve a significant movement of the tip. Conversely, if the instrument has been introduced into the abdomen only a small amount, the handle must move a significant amount to achieve minimal movement of the tip. Consequently many of the motions learned in open surgery, are not applicable to minimally invasive surgery. The surgeon must therefore practice with their assistant in order to achieve the skills necessary for fine instrument movement within the abdominal cavity. Many companies have recognized the challenges associated with intra-abdominal manipulation of instruments, and have worked to develop instruments that simplify movements within the abdominal cavity. This is especially important in terms of hemostasis, suturing, and surgical manipulation within the abdomen.

## 3. Principles of Triangulation

 Minimally invasive surgery requires the use of triangulation. Triangulation occurs when 2 or more instruments are placed through the body wall and move towards the area of interest. When instruments are too close to each other, or too far from each other, it makes surgical manipulation more difficult. If possible, it is best to have the telescope and instruments separated by the appropriate distance to allow the instruments and telescope to converge on the area of interest at an angle of between 30 and 60 degrees.

## 4. Laparoscopy Instruments

### 4.1. Telescopes

 Telescopes used in equine laparoscopy are typically of the rigid Hopkins type. These telescopes comprised of a channel of lenses, and a fiber-optic light channel. They are available either with a 0° forward viewing lens or a 30° for viewing lens. Each of the options has some benefits. The 0° lens allows the surgeon to look straightforward and is often easier to manipulate in the early stages of learning minimally invasive surgery. The 30° forward viewing lens allows the surgeon to have a greater field of view, which provides the opportunity to see more of the abdomen from a single cannula site. The telescopes are also available in different lengths with the majority either 33 cm long or between 54 and 57 cm long. Again there are benefits to each scope length. The shorter scope is easier to use in the early stages of the surgeons training period, whereas the longer scope will allow the surgeon to access more areas of the abdomen. In most cases the long strokes can be advanced to the opposite side of the abdomen in order to do a quick exploratory examination. Operating scopes are also available where a 3rd channel allows introduction of 3 to 4 mm diameter instruments. These telescopes are especially available for single port laparoscopic biopsies.

### 4.2. Cameras, Image Capture, and Monitors

 Cameras allow the surgeon to display the image of the abdomen for all that are involved in the surgery to participate. While it is possible to look through the lens of the telescope without a camera, is difficult to perform minimally invasive surgery without a camera. In most cases the surgeon will use a minimum of 3 portals which requires an assistant to manipulate either the telescope or one of the instruments. If the assistant cannot see what is present in the abdominal cavity it is very difficult for them to assist. It is also much easier to maintain aseptic technique when using a camera. The cameras can easily be connected to image recording devices. These devices are available either as complex integrated systems that work with each individual camera system, or relatively straightforward digital video recording devices. The complex integrated systems are activated by the camera buttons. The other devices can be activated using remote controls that are placed into sterile zip lock bags. The camera will also need to be attached to a monitor. There are many different monitor types available for minimally invasive surgery. Is possible to use a good quality television for the surgeon can purchase a surgical grade monitor. Another alternative to a single monitor is the use of video goggles. There are several commercially available sources for goggles that can be fed a video signal from the endoscopy camera unit. The authors have used goggles manufactured by MyVu (70 Walnut St, Ste 220, Wellesley, MA 02481, http://www.myvue.com/) with good success. Video goggles can be very useful if doing laparoscopic surgery outdoors.

### 4.3. Light Sources

 When performing minimally invasive surgery the surgeon must have light to the abdominal cavity. In equine surgery a 300 W xenon light source is desired. If the light source does not provide adequate light, the telescope must be placed very close to the area of interest. This makes it difficult to find the other instruments, and is very frustrating to the surgeon. The light cord that attaches the light source to the telescope should be the largest diameter light cord available. The larger diameter like chords allow more light to travel to the telescope then the smaller diameter like courts. It is often necessary to have a longer like cord when performing equine laparoscopy as it is difficult to get the light source as close to the telescope as when performing arthroscopy. Over time the fibers in the fiber-optic light cord will break and the amount of available light will be reduced. When the light cord no longer delivers the amount of necessary light, it should be replaced. It should be kept in reserve to be used if the new light cord is damaged. They can also be used for arthroscopy.

### 4.4. Insufflation

 In order to perform minimally invasive surgery the surgeon must create a space to work in [13]. There been many different methods used to create this pneumoperitoneum; including commercially available insufflators, custom-designed insufflators, or by simply using a pressurized canister of carbon dioxide to maintain a working space. The easiest way to maintain working space is by use of an electronic carbon dioxide insufflator. They are designed to create a working space in the abdomen with a consistent intra-abdominal pressure. The value of the consistent pressure is threefold. First, it provides a consistent sized working space for the surgeon that does not require an additional staff member to monitor. Secondly, by maintaining the appropriate pressure, it reduces the possibility of secondary complications associated with high intra-abdominal restaurant. Thirdly, when performing standing laparoscopy, consistent pressures attend to minimize animal movement that occurs with high pressures followed by slowly reducing pressures achieve with simply using a pressurized canister. As more used electronic insufflators are made available, the price has become more reasonable. In man and animals, the recommended intra-abdominal pressure during laparoscopy is 10–15 mm Hg. Pressures greater than 20 mm Hg for prolonged periods can produce negative cardiovascular and respiratory effects and cause some reduction in blood supply to the serosa of the intestinal tract [14]. Traditionally, in domestic animals, CO_2_ is used for insufflation and to create pneumoperitoneum. In humans, a few studies have been performed comparing CO_2_ with room air pneumoperitoneum. In one large study, patients undergoing room air pneumoperitoneum were more likely to have wound infection and abdominal discomfort. However, their conclusion was that room air pneumoperitoneum was safe, cheap, and available and could be used in low resource settings [15]. A more recent study comparing CO_2_ and room air in both laparoscopy and natural orifice transluminal endoscopic surgery found it acceptable [16]. In the author's opinion, in the hospital setting it is easier to use compressed CO_2_ as the tanks are smaller and easier to handle than larger air compressors, and there is no noise produced when they are used. Due to the potential respiratory complications associated with laparoscopy during general anesthesia, intubation and assisted ventilation are recommended in humans and veterinary species. 

 Various insufflation cannulas have been used in equine laparoscopy. The traditional Veress needle can be used for animals that have been placed in dorsal recumbency, but are generally considered to be too short for the flank of horses where standing laparoscopy is performed. The other problem with the Veress needle is that is has a very small diameter, which slows the flow of insufflation gas into the abdomen. The most common early insufflation cannulas for horses in dorsal recumbency has been the “teat cannula” [17–19]. More recently, more surgeons are choosing to perform an open, “Hasson” technique where a laparoscopic cannula with a blunt obturator is introduced into the peritoneal cavity. The main benefits of this technique are that the surgeon only has to place one cannula which saves at least one step, but the cannula also allows the surgeon to insert the telescope prior to insufflation to make sure that the cannula has entered into the peritoneal space. For standing flank laparoscopy, insufflation has been achieved by small diameter chest tubes, mare urinary catheters and more recently laparoscopic cannulas with blunt obturators. The flank is more challenging to approach from an insufflation perspective as the thickness of the flank is greater than that of the body wall at the umbilicus, there are more tissue layers of varying consistencies, and the peritoneum is more loosely attached. There were some complications associated with the sharp trochar catheter placement with bowel puncture and this method has been abandoned [20, 21]. The main benefit of using the laparoscopic cannula and blunt obturator is that the obturator can be replaced with the telescope prior to insufflation to confirm presence in the peritoneal space. 

### 4.5. Trocar's

 Trochars are used in minimally invasive surgery to allow transfer of instrumentation into the peritoneal space without the loss of pneumoperitoneum. The trochar is made up of two distinct units, a cannula and an obturator. Trochars can be reusable or single use in design. Reusable trochars are generally made of stainless steel while single use trochars are generally made of plastic or polyurethane.

 Cannulas also have a number of separate components. The portion that is inserted into the body wall is a thin-walled cannulated sheath that protects the tissues of the body wall from trauma during instrument insertion and exchange, and in some cases provide and avenue for the removal of specimens from the peritoneal space. There are various lengths of cannula sheaths, but the most common are 10 cm (primarily used in human and small animal laparoscopy, or in horses that are placed in dorsal recumbency), 15 cm (primarily used in horses, or bariatric human patients), or 20 cm (primarily used in horses). The longer cannula sheaths allow the surgeon to penetrate greater thicknesses of body wall. However, cannula sheaths that are much longer than necessary require the use of longer instruments, and can be challenging to use for the novice surgeon. Cannula sheaths also come in various diameters in order to allow the insertion or use of different diameter instruments, as well as to allow for removal of larger diameter specimens. The most commonly available sheath diameters are 5 mm (primarily used in human and small animal laparoscopy), 10 or 11 mm (primarily reusable cannulas used in both humans and animals), 5–12 mm (variable use cannulas often used in humans), 12 or 15 mm (used for introduction of stapling devices), and larger diameter (up to 40 mm) for use in specimen removal or specialty surgery such as nephrosplenic space ablation. The cannulas also have a valve the permits insertion of instruments without the loss of pneumoperitoneum. Most cannulas will have a stop-cock that allows the introduction of insufflation gas. The valves generally have two parts; one that maintains intra-abdominal pressure when there are no instruments in place (either a ball valve or a flap valve), and one that maintains intra-abdominal pressure when there is an instrument in place (generally a silicone gasket that has a predetermined size hole in the gasket that seals around the instrument when it is in place). There are many companies that make reusable cannulas, but only a couple of companies that make cannulas with a sheath length of 20 cm. (Surgical Direct of DeLand FL and Karl Storz Endoscopy America of Goleta CA) Surgical Direct actually makes cannulas that allow interchangeable lengths of cannula sheaths. These can be very cost effective for the surgeon. The major benefit of the reusable cannulas is that once purchased they can be used for an indefinite number of times.

 Single use cannulas are available from many companies and can be purchased in many different diameters and lengths. Single use cannulas have the same characteristics as the reusable cannulas. The major difference is the ability to use the same cannula for instruments of various diameters. Many companies manufacture cannulas that will accept instruments from 5 to 12 mm in diameter. This can be very helpful when using 5 and 10 mm instruments along with stapling devices. The valve that seals along he instrument is very tough and flexible so that it can withstand multiple exchanges of instruments. The downsides to single use cannulas is that they are very difficult to resterilize, the gaskets can not withstand multiple sterilizations and uses, and they are relatively expensive. 

In general, the length of the cannula sheath will be determined by the thickness of the body wall of the animal needing surgery. The diameter of the sheath will be determined by the diameter of the necessary instruments. Whenever staplers of any type are used, it is wise to check them against the size of the available cannulas to make sure they are large enough.

 Obturators are placed within the cannulas to allow entry into the peritoneal space. The obturator configuration can be blunt rounded, blunt conical, sharp pyramidal, sharp conical, protected, or optical. The obturator shape is selected based upon the characteristics of the tissue that will be traversed by the cannula, and the possibility of trauma to the underlying structures. The sharp cannulas are more likely to lacerate blood vessels and penetrate intra-abdominal structures that blunt obturators. In general, the author prefers to use blunt obturators for all cannula placements. However, if direct observation of the cannula introduction is possible, sharp obturators can also be used. A benefit of the single use trochars is the concept of a guarded trochar. These trochars are designed to deploy a sharp cutting blade in the obturator during trochar advancement through the body wall, yet, the blade is locked back, or a guard covers the blade, as soon as the peritoneum is penetrated. This feature limits the possibility of injury to deeper structures. There is a reusable guarded trochar available from Dr. Fritz LLC in Louisville Kentucky. The diameter and length of the obturator must be correlated to the similar dimensions of the cannula. 

### 4.6. Hand Instruments

 As the popularity of equine laparoscopy has increased, so have the options for instrumentation. Most of the instruments in human laparoscopy have a working length of 33 cm and are available in either 5 mm or 10 mm diameter shafts. In equine laparoscopy, the 33 cm instruments were either too short, or just long enough for the surgical procedure. As the human population has increased in body size with obesity, and as companies have become more interested in developing equine instruments, 45 cm working length instruments are now available. The extended length provides opportunities to reach structures deeper in the abdomen, but increases the challenge faced with the fulcrum effect of the body wall. Most equine surgeons would like to have a set of normal length instruments as well as extended length instruments, but this is not often practical. Most of the instruments that have been designed for open surgery are currently available for laparoscopic surgery. The major difference is that the instruments for laparoscopy are longer, have smaller jaws, and do not have inline handles. Most reusable instruments are designed to be taken apart so that they can be carefully cleaned. It is important to recognize that cannulated instruments, and instruments with complex, articulated, components have the ability to house infectious agents unless very thorough cleaning and sterilization processes are followed. In one study of arthroscopic instruments in a Texas hospital, there were 7 infections following knee arthroscopy over a two week time span [22]. The conclusion of the review was that the infections were likely related to surgical instrument contamination with *P. aeruginosa* during instrument reprocessing. It was possible that retained tissue in inflow/outflow cannulae and shaver handpieces could have allowed bacteria to survive sterilization procedures. To this end, companies such as Medisafe America (Tampa, FL) have developed sonic cleaners that not only offer ultrasonic cleaning of the outside of the instruments, but also send jets of ultrasonic fluid through the lumens of cannulated instruments as well. Resterilization of single use instruments can be performed, but is done at the risk of the surgeon and the hospital.

 Hand instruments are available as dissecting instruments, grasping instruments, and scissors. Many different configurations are available. Many of the instruments can be purchased with a monopolar electrosurgical connection that is more completely described in one of the following sections. The handles can be purchased with locking mechanisms if desired. It is important to have strong, traumatic, grasping forceps when performing equine ovariectomy. These instruments help to stabilize the ovary during manipulation and transection, but more importantly during removal from the abdominal cavity.

 Needle holders are also available for minimally invasive surgery. The traditional needle holders have a 33 cm working length and a 5 mm diameter shaft. They are available with in-line handles, or handles similar to the other hand instruments. The traditional needle holders work well in small animal laparoscopy, camelid laparoscopy, and in foals. However the jaw configuration is such that only the smaller needles can be held securely. For the larger needles used in surgeries such as nephrosplenic space ablation, it is better to have needle holders with larger jaws. Surgical Direct in Deland, FL makes a set of needle holders that have a 40 cm working length and are 10 mm in diameter. They are designed to be used with large needles such as those found on size 0 or 1 Maxon (Polyglyconate, Covidien, Mansfield, MS) or size 2 Vicryl (Polylactin 910, Ethicon, Summerville, NJ).

## 5. Miscellaneous Instruments

 Depending on the type of surgery performed, or the needs of the procedure, other instruments can be useful. These specialized instruments include, but are not limited to, injection needs, knot pushers, and reducing cannulas. 

Injection needles are very valuable for providing local anesthesia to the mesovarium, mesorchium, or other structures that need to be manipulated during standing surgical procedures. They can also be valuable to inject local anesthetic into structures that will have tension applied while the animal is under general anesthesia, both to reduce the amount of general anesthetic necessary as well as to improve pain control post operatively. Conversely, the injection needles can function as suction needles to remove fluid from cystic lesions, granulosa thecal cell tumors, potential abscesses, and hematomas to name a few indications. Reducing the volume/size of structures prior to removal from the abdominal cavity can reduce the size of the incision in the body wall.

Knot pushers have been developed to tighten knots that are tied extracorporeally and placed/pushed into the abdomen. These knots can be tied as ligating loops and passed through the cannula into the abdomen, or a knot can be tied one throw at-a-time and pushed through the cannula into the abdomen. They can have completely cannulated lumens, or may only have a slit at the end where the suture is tensioned. Knot pushers must be long enough to easily penetrate the longest cannula used in the surgical procedure. If the tip of the knot pusher does not penetrate past the end of the cannula, knot security will be reduced or nonexistent. When knot pushers and ligating loops are used, a knot protector should be used as well. Knot protectors allow the ligating loop to be pulled inside of the protector so that when the protector is placed through the valves in the cannula that the ligating loop is not disturbed. 

## 6. Ligation and Hemostasis Techniques

 Effective ligation of structures within the abdominal cavity and hemostasis are critical steps in minimally invasive surgery. Because of the challenges of the two-dimensional viewing and fulcrum of fact of the body wall, many different techniques have been developed to provide hemostasis and ligation. These techniques include: ligature placement, sharp dissection and ligature placement, polyamide tie-raps, monopolar and bipolar electrosurgery, vessel sealing devices, ultrasonic devices, surgical staplers, and laser dissection. While all of these methods have proven efficacious, certain advantages and disadvantages relating to surgeon preference, equipment costs, speed, reliability, and technical difficulty exist. 

### 6.1. Ligating Loops

Ligating loops are one of the first techniques that were developed for intra-abdominal ligation and hemostasis. In general, they are the least expensive method of hemostasis used during laparoscopic surgery. This is especially true when self-tied ligating loops are used. By getting loops are available commercial ready, and can be self tied during the surgical procedure. The challenge of using ligating loops is that they are more technically demanding than using some of the other methods of hemostasis.

The first reports on using ligating loops in horses for castration was in 1996 where Wilson et al. [23]. retracted normally descended testes back into the abdomen and ligated them using commercially available ligating loops. Bouré et al. [24] used commercially available ligating loops to ligate and provide hemostasis during ovariectomy in mares. In this study, there was no dissection of the ovarian pedicle until after placement of two ligatures. When they sharply transected the pedicle, it was between the ligatures. There were no complications noted with bleeding in these cases. A benefit of commercially available like getting loops is that the hopes are tied, and then sterilized, to maintain an open loop. One of the early concerns with commercially available ligating loops was the fixed diameter of the loop, the small size of the available suture, and the short knot pusher that was attached to the ligating loop. Several self-tied knots have been developed to allow the surgeon to make their own ligating loops patient side. Two studies on the use of larger sutures for horses confirmed the ability to self-tied ligating loops in order to provide equal or better knot security when compared to commercially available ligating loops [25, 26]. These studies identified the importance of combining and appropriate knot with the appropriate suture material. The challenge of the studies is that no one knows how secure the knot must be in order to minimize bleeding after placement. Until the necessary tension can be identified, or what knot security needs to be, surgeons will undoubtedly continue to use the most secure material possible. The studies also highlighted the ability of monofilament suture material to plastic lead to form upon tightening the knot providing increased not security over coated, braided suture material. In some cases, especially when using the 4-S Modified Roeder knot with Size 1 Maxon, the knot security approaches that of a four-throw square knot. While monofilament sutures provide greater knot security, the inherent memory can cause the loop to twist after being tied. The smallest diameter of suture material should be used so as to limit the amount of foreign material within the abdomen.

However, ligating loops can slip after application [27]. This can be due to not getting them tight enough for the knot to lock when placing the loop, having too much tissue in the loop, cutting the tissue too close to the loop during transection, or by placing the loop on a wedge shaped piece of tissue. Rodgerson reported ligating loop slippage after an ovariectomy, and the author has had a ligating loop slip during cryptorchid castration. It appears important to relax the tissue when finally tightening the loop to minimize tension on the tissue to allow for secure knot locking. 

In summary, ligating loops have been consistently shown to be adequate for ligation and hemostasis in equine laparoscopy, especially in the areas of ovariectomy and cryptorchidectomy. The author prefers monofilament suture material both due to the increased knot security over braided material, as well the propensity of the loop to hold its shape during placement.

## 7. Polyamide Tie-Rap

A challenge for the surgeons using ligating loops is to position the loop around the desired structure. Polyamide tie-raps hold their shape better than sutures, and can be used to lie gate structures in a similar fashion. A study on the use of a commercially available polyamide tie-raps showed that the tie-raps can be successfully used to ligate and provide hemostasis in equine ovariectomies [28]. In the study, 10 horses were ovariectomized using the tie-raps. Repeat laparoscopy was performed in 8 of 10 mares, 2, 3, 4, and 12 weeks post ligation. The transected stump was completely encapsulated by 3-4 weeks. In two mares, an adhesion between the left stump and the mesentery of the descending colon was observed. There were no remarks as to the consequences of the adhesions. The author has had to reoperate one mare that had clinically significant adhesions between the stump and the mesocolon after ligation with a polyamide tie-rap. The adhesions were successfully transected and the horse has shown no more signs of abdominal pain.

In summary, the tie-raps can be used in ovariectomy, but the occurrence of adhesions is troubling, and brings the technique into question.

## 8. Monopolar and Bipolar Electrosurgical**** Devices

Monopolar and bipolar electrosurgery provide opportunities for hemostasis in the abdominal cavity for removal of cryptorchid testes, descended testes, ovariectomy, as well as adhesiolysis [23, 29–31]. Monopolar electrosurgery can be delivered via many configurations of laparoscopic instruments, requires the use of an electrosurgical generator, an active electrode, and a grounding pad. When using monopolar electrosurgery, the energy passes through the body from the active electrode to the passive electrode, or ground. The effectiveness of the active electrode is determined by the size of the electrode as it comes in contact with the tissue. This safety of the day passive electrode, or ground, is determined as well by the size of the electrode. In general, the active electrode should be as small as possible, and the passive electrode should be as large as possible. In this way, the energy is focused at the small electrode, and spread over a large area with a large electrode. Monopolar electrosurgery can be used in either the cut, coagulate, or blend modes. When using thick cut mode, the tissues are divided by focusing the electrical current over a small area causing vaporization or explosion of the cells, ideally in a noncontact method. When the active electrode contacts the tissue, the current is less focused leading to a desiccation of cellular water and thereby a coagulum. After cell vaporization in noncontact mode, the heat is dissipated, thereby minimizing heat transfer to deeper tissues. In this instance, coagulation and hemostasis are minimized. In contact mode the thermal damage occurs over a greater area improving hemostasis. The tissue effects are controlled by the generator output. In most cases the heat generated at the tip of the active electrode is approximately 150°C leading to tissue damage bed is 2 to 3 mm deep and 2.5 to 3 mm lateral. Monopolar electrosurgery is considered to provide good cutting and coagulating a cavity. However coagulation is limited to vessels 3 mm in diameter or smaller [32].

 Monopolar electrosurgery is likely the most commonly used electrosurgical modality in human laparoscopy [33, 34]. One of the main reasons multiple electrosurgery is so popular in human laparoscopy is associated with the minimal cost experienced by the surgeon. The majority of surgical practices already on a monopolar electrosurgical generator and many available instruments have attachments that allow monopolar electrosurgery. A popular way to use monopolar electrosurgery is by attaching the wiring from the generator to scissors so that the operator can coagulate at the same time as cutting. The main concern with monopolar electrosurgery is that it is possible for energy to leave the instrument and return to the generator through surrounding soft tissues. Therefore is is important for the operator to minimize any possibility of stray energy impacting tissue that is not supposed to be involved. It is also important to minimize contact of the active electrode with shielded structures such as cannulas, telescope, and other instruments. Monopolar electrosurgery has been used in combination with other ligating techniques such as ligating loops.

Bipolar electrosurgery is delivered via an instrument that clamps the desired tissue between the electrical energy used in bipolar electrosurgery travels only between the two jaws. Consequently, bipolar is thought to be safer than monopolar with regard to stray energy affecting other tissue. The heat generation, tissue damage, and coagulating ability are similar to monopolar electrosurgery however the cutting ability is considered to be poor. The reports have shown that bipolar electrosurgery can be successfully used in both ovariectomy and cryptorchidectomy. In most cases, sequential application of the electrosurgical device and sharp dissection have been used [29, 35]. Some bipolar instruments incorporate both the electrosurgical paddles as well as a cutting blade, reducing the need to exchange instruments during the procedure. The main limitation to bipolar electrosurgery appears to be in the quality and capability of the generator.

 In summary, these modalities are easy to apply to the horse. Both mono- and bipolar electrosurgery can be performed in the standing sedated horse. While successful, it should be noted that these modalities are recommended for 3 mm diameter vessels or smaller. Care should be taken in cases where the vessels are larger than 3 mm. It is also important to note that hemostasis is accomplished by forming a coagulum in the vessel lumen that can be dislodged with movement or increased blood pressure.

## 9. Vessel Sealing Devices

 The reduced vessel size capabilities of mono- and bipolar electrosurgery have lead to the development of other, more sophisticated, electrosurgical devices. These devices are generally known as vessel sealing devices, and use radio-frequency energy in a bipolar fashion to create sealed vessels as compared to a coagulum in other electrosurgical techniques. The bipolar electrodes are essentially feedback controlled electrothermal sealers that apply a precise amount of energy to vessel walls to produce a denatured protein seal. The most commonly used device in horses is the LigaSure from Valley Lab. (LigaSure, Valley Lab/Covidien, Boulder, CO) It is designed to seal vessels up to 7 mm in diameter and withstand blood pressures up to 3 times the normal. The shears generally incorporate a cutting blade so the instrument can be used to coagulate and cut sequentially without removing or exchanging the instrument.

 The use of vessel sealing devices have been reported in horses for the removal of normal ovaries as well as removal of granulosa theca cell tumors [36–38]. In most instances, the hemostasis is complete with no bleeding at any point in the procedure. There have been no reported complications. There does appear to be a significant increase in total protein in the abdominal fluid at 24 hours post-surgery when comparing the vessel-sealing device to the use of ligating loops for ovariectomy. The total protein returns to a similar level to the ligating loop by 72 hours. The clinical significance is not known. Vessel sealing devices have also been used for cryptorchidectomies and adhesiolysis.

In summary, the vessel-sealing devices are generally accepted to be the most secure, easy to use device for removal of ovaries and testes in the horse. The ability to seal a 7 mm vessel is quite valuable in the hemostasis and amputation of large ovaries. The only downside is the cost of the generator and the shears, which are meant to be disposable.

## 10. Ultrasonic Cutting and Coagulating Devices

 Ultrasonic cutting and coagulating devices rely on sound waves to cause intended tissue effects. At high power levels and density, the sound waves can be used for tissue dissection, coagulation, and cutting. Ultrasonic surgical units consist of a generator, foot switch, and hand piece. In most surgical units, the hand piece consists of a transducer and some type of blade system that vibrates at a frequency of 55,500 cycles per second at a distance of 50 to 100 microns per cycle. The blade configuration determines the tissue effect, where shaper blades will cut with less coagulation and blunt blades will cut more slowly while providing better coagulation. Some instruments are configured with jaws at the end where one portion of the jaw holds the tissue still, and the other jaw is active. The generator power level along with the blade type will determine the amount of energy delivered to the tissue. The amount of blade tissue contact, pressure, blade speed, and activation time will determine the tissue response to the energy delivered. Tissue coagulation occurs when the mechanical energy of the blade is transferred to the tissue, breaking tertiary hydrogen bonds. The friction and shear stress caused generate a moderate amount of heat, in combination with the breaking of hydrogen bonds leads to sticky protein coagulum. If more pressure is placed on the tissue, and the power setting increased, cutting occurs. The heat generated is generally less than 150°C, with 0.5–2.0 mm depth and 0.2–3 mm lateral tissue damage. The cutting and coagulating abilities are both considered good. Vessels up to 3 mm in diameter are thought to be reasonable for coagulation. One of the major benefits of ultrasonic surgery over electrosurgery is that there is no potential for passing electricity through the patient. However, the tip of the blade can remain hot for a few minutes after activation. Consequently, the jaws should always be kept in the field of view and should not come in contact with normal tissue.

A benefit of ultrasonic cutting/coagulating devices in surgery is that hemostasis and transection can be performed with the same instrument, obviating the need to switch instruments. Benefits include potentially faster surgery time and reduced chance of infection. The only published reports in horses have been for ovariectomy of normal ovaries. In one study involving 8 mares, all 16 ovaries were removed without any bleeding [39]. In the other study involving 10 mares, 40% of the ovarian pedicles required additional hemostasis in the form of ligating clips. The overall concern described in the paper is that the second setting the energy setting was too high, favoring rapid transection without complete hemostasis [40]. While no published reports are available, the author has used the ultrasonic device for successful cryptorchidectomy. These devices have been shown to be very useful in adhesiolysis in human surgery, especially when using the blunt blades [41].

 In summary, the ultrasonic devices are designed to coagulate vessels up to 3 mm in diameter. The equine ovarian pedicle can sometimes contain vessels that are larger. Methods of additional hemostasis should be available when using these devices for equine ovariectomy. It is not recommended to use these devices for granulosa theca cell tumors. 

## 11. Stapling Devices

 Surgical stapling devices have been developed to allow easy and safe hemostasis and amputation of abdominal structures. In one study involving 10 horses undergoing laparoscopic ovariectomy, the endoscopic stapling device was successfully used to ligate and amputate normal ovaries [42]. The surgical time was thought to be less than that with ligating loops, required minimal ovarian manipulation, and there were no intra, or postoperative complications. Endoscopic surgical staplers require larger diameter cannulas than are typically used in equine laparoscopy, and the surgeon should be prepared for this before attempting to use surgical staplers. Traditional staplers have been used for hand assisted ovariectomies of large granulosa theca cell tumors [43]. An incision that is large enough for the surgeon's hand and telescope is made in the flank on the side of the granulosa theca cell tumor. The pedicle is desensitized with local anesthetic and the stapler is advanced adjacent to the surgeon's arm and applied. The benefit of this technique is that the traditional staplers are generally less expensive to use than the endoscopic staplers.

 In summary, surgical staplers are technically easy to use and very effective in providing hemostasis for equine ovariectomy. The biggest problem is the cost of the stapling devices and the need for a larger cannula when using endoscopic stapling devices.

## 12. Surgical Lasers

 Laser surgery is performed using light amplification of various substances to create heat. The laser beam consists of photons that exit the amplification chamber in specific wavelengths based upon the lasing medium. Commonly available lasing mediums in veterinary medicine include CO_2_ and diode semiconductors. CO_2_ lasers are more efficient cutting lasers, and provide minimal collateral damage. CO_2_ lasers are often considered as “what you see is what you get” lasers in that the tissue damage that is seen at surgery is similar to what will be seen in the post-operative period. Diode lasers are less efficient cutting lasers, and will have up to 3 mm collateral damage to the tissue. CO_2_ laser energy is delivered through semirigid wave guides or articulated arms making them best suited for skin or open surgical techniques. New wave guides may be available to offer minimally invasive delivery in the future. Diode laser energy is delivered through a crystal fiber, allowing introduction into cavities through cannulas or flexible endoscopes [44].

 Surgical lasers have been used to provide hemostasis in both open and laparoscopic surgery. There is one report of the use of lasers and endoscopic stapling devices in unilateral ovariectomy in normal mares [45]. There were no surgical complications, and the mares resumed normal activity 2 weeks after surgery. There is some concern that lasers will not provide appropriate hemostasis when used to amputate large ovaries with larger diameter vessels. Lasers are generally considered to be capable of providing hemostasis in vessels that are 3 mm diameter or less. Additional methods of hemostasis, such as ligating clips or surgical staplers, should be available when using lasers for equine ovariectomy.

## 13. Sedation and Anesthesia

### 13.1. Standing Procedures

One of the main benefits of laparoscopic surgery in the horse has been the ability to do the procedure from a standing position. This reduces the need to anesthetize the horse along with all of the complications and expense that accompany general anesthesia. However, it is important to have the horse stand as still as possible in order to limit the possibility of complications during surgery. In order to perform standing surgery, a combination of sedation along with local anesthetic is necessary. 

### 13.2. Sedation

There are a number of options available for sedating horses. The most common sedation drugs include alpha-2 agonists and opioids. Drug combinations include xylazine (0.5–1.0 mg/kg) and butorphanol (0.02 mg/kg) or detomidine (2–10 ug/kg) and butorphanol (0.02 mg/kg). Owing to the length of the typical standing procedure, many surgeons use an option that will provide a longer lasting sedation than that achieved with a single dose of intravenous sedation. Sedation has been accomplished using alpha-2 drugs in a caudal epidural as well as with a continuous rate infusion of alpha-2 drugs. One study evaluated the use of detomidine and buprenorphine in combination for sedation in standing laparoscopic surgery [46]. The sedation was antagonized after surgery using atipamezole. There were no changes in measured parameters when compared to a control group that was sedated without undergoing surgery. 

### 13.3. Epidural

A xylazine epidural can be performed at the S5-C1 or C1-C2 space (0.18 mg/kg xylazine qs to 10 mL with saline) [47, 48]. In one surgeon's experience, this approach allows ovarian manipulation without instilling local anesthetic into the ovarian pedicle in approximately 50% of horses. The author feels that the manipulation of the ovary or cryptorchid testis is not consistently tolerated. Consequently the author recommends using local anesthetic in the ovarian pedicle and the mesorchium prior to manipulation. Detomidine epidurals have also been used (40–60 Mg/kg qs to 10 mL with saline). The reported dose is 60 Mg/kg, but the author has experienced severe ataxia in horses, with one becoming recumbent using the higher dose, and chooses to use a dose of no more than 40 Mg/kg for surgery [49]. When detomidine is used in the epidural space, the caudal abdomen is often desensitized. It is important to realize that using detomidine as an epidural will provide significant sedation requiring dose modification of any supplemental systemic sedation protocol.

### 13.4. Continuous Infusion

Medetomidine and morphine have been evaluated for their behavioral and cardiorespiratory effects when used for standing laparoscopy. The continuous infusion of both drugs enhanced sedation and ataxia. It was felt that the combination resulted in reliable sedation and stable cardiorespiratory function [50]. A continuous rate infusion of detomidine supplemented with butorphenol has been used for sedation [51]. The author uses 20 mg of detomidine in a 1 liter bag of polyionic replacement fluids. The drip rate is determined by the surgeon, and can be increased or decreased as needed for the appropriate plane of anesthesia. In a recently completed (unpublished) study, comparing continuous infusion to epidural administration, there was no statistical difference in the two techniques, but there was a trend for better sedation, and less response to stimuli with epidural administration. However, the use of a continuous infusion was found to be easier to perform and very well tolerated by the horse. 

### 13.5. Complications with Sedation

 The reported dosage for epidural detomidine was 60 mcg/kg [48]. One report suggested that this dose was too high and described a horse that became very sedated, unstable, and eventually fell down [49]. If the horse becomes too sedate and unsteady, surgical stimuli may be enough to improve the horse's depth of sedation. However, it must be noted that if you have already placed instruments, and the horse collapses, it can be very detrimental to your instruments. In general, it is better to give the horse some time to recover prior to placing portals and instruments. Not every horse is compatible with standing surgery under sedation and local anesthesia. It is important when looking at patient selection to be as sure as possible that the horse will stand in the stocks for the procedure. In the author's experience, the most likely times for the horse to jump out of the stocks include epidural placement, portal placement, and grasping the testis or ovary prior to local anesthetic blockade. It is worthwhile to ask the owner if the horse tolerates being tied, being placed in stocks, and being injected. If the horse is very difficult to inject, standing surgery is probably not the best idea.

### 13.6. Body Wall Local Anesthesia

Local anesthesia of the skin and body wall can be accomplished with an inverted “L” in the paralumbar fossa, a paravertebral block, or by instilling local anesthetic at each portal site. The author prefers to use 2% lidocaine at each portal site using a 20 gauge 1.5 inch needle. It is important to remember when using portal site anesthesia that the skin blebs must be visible. If the skin is incised distant to the block, the horse is likely to respond quite negatively. It is possible to use too much lidocaine in horses. While the critical dose is not completely understood, the author chooses not to exceed 200 mL per 500 kg horse. During standing surgery, the patients are only sedated and will often move during the procedure. They also maintain skin sensation. It is possible to deposit local anesthetic where the towel clamps will go, but it is generally difficult to find the “blebs” under the drapes. The author chooses to not use local anesthetic, but rather to apply the towel clamps very slowly to limit response of the horse. Another option is to use a skin stapler to attach the drape to the horse [52]. This is another opportunity for the horse to jump out of the stocks. While not proven, it is the author's opinion that using a caudal epidural with detomidine for sedation will reduce the responsiveness of horses to towel clamp placement when compared to sedation by intravenous techniques.

### 13.7. Intra-Abdominal Local Anesthesia

 While some types of sedation will limit the movement of the horse when performing intra-abdominal surgery such as for ovariectomy or cryptorchidectomy, it is not consistent. The author would recommend the use of 2% lidocaine in the mesovarium and mesorchium prior to ligation and amputation. A series of studies have shown the benefit of instilling local anesthetic in these regions to limit movement of the horse [53, 54]. Injection directly into the ovary is less effective, and injection into the testis enlarges the testis and requires more time for the local anesthetic to take effect. In one study involving foals, it was determined that the epidural cranial migration of local anesthetic is dependent on the volume of the injectate, not the positioning [55]. An epidural injection of 2% mepivacaine could provide analgesia up to at least the caudal thoracic dermatome.

### 13.8. Patient Positioning

 Patient positioning is important in equine minimally invasive surgery. The bowel, especially the large colon and cecum, is generally too heavy in the adult horse for meaningful manipulation. Consequently, the surgeon is only able to observe what is “on top” as the large bowel obscures deeper structures. If dorsal abdominal structures are of importance, then a standing surgery is preferred, if ventral abdominal structures were of importance, then a dorsally recumbent surgery would be preferred. If dorsal structures are involved and can be lateralized, left or right sided, and the horse is not a candidate for standing surgery, the horse can be placed in lateral recumbency with the affected side “up”.

## 14. Anatomy

### 14.1. Standing Anatomy

 The body wall of the standing horse is comprised of skin, subcutaneous tissue, external abdominal oblique muscle and fascia, internal abdominal oblique muscle, transverse abdominus muscle, and peritoneum. The landmarks are the caudal ribs, the transverse processes of the vertebra, and the tuber coxae. The vessels of concern are the circumflex iliac artery and vein which are located near the dorsal border of the internal abdominal oblique muscle [20]. Common structures visible in the standing horse from the left flank include: stomach, diaphragm, spleen, kidney (retroperitoneal), small colon, mesocolon, small intestine, left ovary in mares, left uterine horn in mares, left vaginal ring in males, bladder, and whatever part of the colon that is dorsal. From the right flank the following structures are generally visible: duodenum, epiploic foramen, ventral band of the cecum, small colon, mesocolon, small intestine, right ovary in mares, right uterine horn in mares, right vaginal ring in males, bladder, and whatever part of the colon that is dorsal [56].

### 14.2. Dorsally Recumbent Anatomy

 The body wall of the dorsally recumbent horse is comprised of skin, subcutaneous tissue, external rectus sheath, rectus abdominus muscle, internal rectus sheath, linea alba, and peritoneum. The landmarks are the umbilicus, the ziphoid process, and the inguinal canals. The vessels of concern are the superficial and deep caudal epigastric arteries and veins which are found at the abaxial portion of the rectus abdominus muscles. Common structures visible in dorsal recumbency are liver, diaphragm, bladder, spleen, small intestine, and whatever part of the colon and cecum is on “top” [57, 58]. The caudal abdomen is best observed with the animal in Trendelenberg position (head down and tail elevated) while the cranial abdomen is most easily observed with the animal in Reverse-Trendelenberg position (head elevated, tail down).

## 15. General Surgical Concepts

### 15.1. Standing Surgery

 The general approach used for standing laparoscopy begins with fasting the horse for 12–24 hours to decrease the volume of the intestinal contents, and consequently improve visualization [59]. The use of nonsteroidal anti-inflammatory drugs and antibiotics is variable. The flank of interest is clipped and aseptically prepared for aseptic surgery. When an exploratory is planned, it is recommended that both flanks be prepared for surgery. Sedation is surgeon's preference with many options available as has been previously described. Single dose intravenous (IV), continuous infusion of IV sedation, IV sedation along with a caudal epidural, or caudal epidural using detomidine alone have all been reported [53, 54]. The skin and musculature of the flank is can be infiltrated with 40 to 60 mL of local anesthetic (2% lidocaine or 2% mepivacaine) in an inverted “L” pattern, or more recently only the portal sites are blocked with 10–15 mL local anesthetic per site. The horse is draped to allow access to one or both flanks as needed. The author prefers to insert either an 8 mm diameter mare urinary catheter, or a 10 mm diameter, 20 cm long cannula with a blunt trochar (Surgical Direct, DeLand, FL) into the peritoneal space through a small stab incision in the skin in the middle of the flank (at the ventral most level of the tuber coxae, midway between the last rib and the tuber coxae) for carbon dioxide insufflation before trochar/cannula placement. Another option is to use a controlled access canula (Ternamian EndoTip Cannula, Karl Storz Veterinary Endoscopy). Presence of the tip of the catheter in the peritoneal space is confirmed with negative pressure and the sound of air being drawn into the abdomen. When using a cannula with a blunt obturator, the telescope can be inserted into the cannula to determine correct placement within the peritoneal space. Once the abdomen is distended, one or more trochar/cannulas are placed through the body wall. A skin incision similar in size to the cannula is made and the trochar/cannula is placed into the abdomen with a slow, but constant, twisting motion. It is generally not necessary to use a sharp obturator to place any of the cannulas. However, after the first cannula is in place, a sharp obturator can be used if the insertion can be directly observed. Sharp obturators increase the possibility of vessel laceration and portal bleeding. The telescope is placed through the dorsal most cannula and connected to the light source and video camera. As many cannulas as necessary, limited of course to the size of the surgical site, can be placed. Cannulas can be placed in the 17th intercostal space. At the end of the procedure, the abdomen is desufflated (passively, or actively with suction), the cannulas removed, and small incisions are closed with simple interrupted skin sutures, while larger incisions are closed with a simple continuous pattern in the external abdominal oblique muscle and fascia, and a simple interrupted pattern in the skin. The most limiting factor in performing standing laparoscopy is the behavior of the horse. The horse must be willing to stand during the procedure without “going down”, or jumping out of the stocks. There is increased risk to the surgeon and equipment during standing laparoscopy, but decreased risk to the patient. There have been changes associated with carbon-dioxide insufflation of the standing horse [60]. Pneumoperitoneum with CO_2_ during standing laparoscopy in healthy horses does not cause adverse alterations in cardiopulmonary, haematology, or plasma chemistry variables, but does induce a mild inflammatory response within the peritoneal cavity.

### 15.2. Laterally Recumbent, Flank Laparoscopy

 Preoperative preparation is similar to that for the standing horse. The horse is anesthetized and placed into lateral recumbency with the desired side of the abdomen facing up. The flank is clipped and aseptically prepared for surgery. Insufflation is performed similarly to the standing flank approaches. This approach only gives access to the uppermost side of the abdomen and is generally reserved for removing enlarged granulosa thecal cell tumors, or for horses where the area of interest is limited to one side of the dorsal abdomen and they are not candidates for standing surgery.

### 15.3. Ventral Midline Laparoscopy

 Preoperative preparation is similar to that for the standing horse. Surgeons often hold the horses off feed for a longer period of time in order to further reduce the ingesta volume [61]. This makes it easier to access dorsal abdominal contents. The horse is anesthetized, placed in dorsal recumbency, and the ventral abdomen is aseptically prepared for surgery. The exact location of the draping depends upon the area of interest, but usually includes the umbilicus and the body wall out to the folds of the flank, and either to the inguinal region or the xiphoid. Insufflation is achieved by inserting a Veress needle, teat cannula, or laparoscopic trochar at the umbilicus and connecting to a carbon dioxide insufflator to distend the abdomen to 10–15 mmHg. Once the abdomen is distended, the veress needle or teat cannula is removed the skin incision increased to the size of the telescope cannula, and a cannula with a blunt obturator is placed through the body wall. The animal can then be tilted so that the rear quarters are elevated (Trendelenberg position) to ease exploration and surgical manipulation in the caudal abdomen, or tilted so that the fore quarters are elevated to ease exploration and surgical manipulation in the cranial abdomen. In either case, the horse should be tied to the table to reduce the possibility of the patient sliding off the table. When placing the horse into Trendelenberg position, it is important to have access to positive pressure ventilation in order to properly ventilate the patient [62, 63]. Other cannulas may be placed under direct visualization. Some surgeons prefer to preplace a spinal needle to determine the exact entry point of the additional cannulas. 

## 16. Surgery of the Male Reproductive Tract

### 16.1. Cryptorchidectomy—Standing

 When performing a standing cryptorchidectomy, the testis or mesorchium should be infiltrated with 10–15 mL local anesthetic [54]. The first report of surgical cryptorchidectomy included exteriorizing the testis from the abdominal cavity and emasculating similar to a normal castration [64]. The testis can be ligated and amputated using any of the previously mentioned techniques [29, 30, 33, 65–68]. If the horse is a bilateral cryptorchid, a long knot pusher (Surgical Direct, DeLand, FL) can be used to allow the surgeon to remove both testes from the left flank. When performing a bilateral cryptorchidectomy from one flank, a fourth cannula is necessary. After amputating the testis/testes, the ventral most incision is enlarged, and the testis/testes are removed. It was originally thought that testes that were not in the abdomen but had not fully descended into the scrotum could be amputated by simply transecting the cord allowing the testes to undergo aseptic necrosis [23]. However, it has subsequently been shown that the testis can revascularize and still produce testosterone [69, 70]. The testis has a complex blood supply that allows the testis to revascularize from vessels in the tunic, especially the cremaster vessels and the external pudendal artery [71].

At our institution, if a descended testis is present, it is removed using a short acting anesthetic agent. However, standing castration is also feasible. While it is rare to identify an abnormally enlarged testis, it is possible for a teratoma to exist [72]. It is the author's opinion that standing laparoscopic cryptorchidectomy is easier to perform than while under general anesthesia since the testis hangs from the dorsal body wall. The animals are generally able to return to athletic function within 3 days following surgery.

### 16.2. Cryptorchidectomy—Dorsal Recumbent

This technique requires only moderate tipping into Trendelenberg position. The testis/testes are almost always located adjacent to the bladder. The telescope is placed at the umbilicus and two or more portals are placed in the parainguinal region [61, 73]. Single ligating loops or a Ligasure device is used to ligate and amputate the testis. This approach is valuable in horses that are going to show or perform within two weeks of surgery since it does not require clipping of the flank hair. If the horse is a bilateral cryptorchid, both testis can be ligated and removed through a single enlarged incision.

### 16.3. Other Male Reproductive Surgery

 Inguinal herniorrhaphy has been described in the horse [23, 74–77]. Fischer et al. described the first inguinal herniorrhaphy in two intact stallions using a retroperitoneal mesh in order to maintain breeding function [74]. Flap hernioplasty was described by Rossignol et al. in 2007 [78]. The group evaluated the approach in 9 normal animals and 4 clinical cases. The flap was evaluated laparoscopically post-surgery and the rings in all cases were deemed to be secure. Caron and Brakenhoff described using intracorporeal suture closure of the internal inguinal and vaginal rings after castration [77]. A retrospective study on a flap hernioplasty technique in 30 horses showed the importance of complete closure of the ring to limit reherniation [79]. Laparoscopy has also been used successfully in horses where hemorrhage exists after castration [80, 81].

## 17. Surgery of the Female Reproductive Tract

### 17.1. Ovariectomy—Standing

When performing a bilateral ovariectomy, both flanks must be prepared for surgery. Six cannulas are necessary for this procedure. The mesovarium should be infiltrated with 10–15 mL of local anesthetic prior to amputation [53]. With normal sized ovaries, the ovarian pedicle can be ligated using ligating loops [24, 30, 82–84], ultrasonic devices [39, 40], vessel sealing devices [30, 36, 37], electrosurgery [31, 35, 85], lasers [45], stapling devices [42], or polyamide tie-raps [28]. When using ligating loops, a shorter knot pusher is used in order to have a larger ligating loop to fit over the ovary. After amputation of the ovaries, the right ovary is passed under the small colon to the left side of the abdomen, and both ovaries are removed from the enlarged ventral flank incision. In juvenile mares, it has been reported that the ovary can be amputated and dropped within the abdomen with no untoward effects [85]. In cases of granulosa thecal cell tumor (GCT) removal, only the flank of the affected ovary need be prepared [38, 86]. It can be difficult to use ligating loops on an enlarged GCT, consequently a Ligasure device is generally recommended. It is possible use a ligating loop on an ovary up to 18 cm in diameter, and to remove an ovary up to 25 cm in diameter using a Ligasure device. If the GCT has large cystic areas on ultrasound, it is possible to use an injection needle along with a suction device to remove cystic fluid thereby reducing the size of the ovary. There have been a number of reports on the use of hand-assisted laparoscopy for ovariectomy [43, 87–89]. While an enlarged incision is necessary for this technique, the eventual size of the incision is not dissimilar to the final incision for ovarian removal. This is especially true in the case of GCT's, and can be technically easier than a straight laparoscopic approach. While granulosa theca cell tumors are the most common disease process with enlarged ovaries, teratomas may also be present [90]. Other techniques for removing large granulosa cell tumors include morcellators [91] and surgical pouches [38]. It is the author's opinion that standing laparoscopic ovariectomy is easier to perform than while under general anesthesia since the testis hangs from the dorsal body wall.

### 17.2. Ovariectomy—Dorsal Recumbent

Dorsally recumbent ovariectomy requires aggressive tipping into Trendelenberg position. It is recommended that the table be tipped at least 30 degrees in order to move the abdominal visceral forward and away from the uterus and ovaries [92, 93]. The ovaries and ovarian pedicles are not as accessible in a dorsally recumbent horse as they are in a standing horse. Two ligating loops per ovary, or a Ligasure device can be used to ligate and amputate the ovaries.

### 17.3. Other Urogenital

 In 1988, laparoscopy was considered an adjunctive method of examining the urogenital tract in horses [94]. It has now become a mainstay in the armamentarium of the equine veterinarian. A report on the use of laparoscopy for the diagnosis of a uterine tear was published as early as 1994 [95]. The tear was managed conservatively after the diagnosis. One report described the use of laparoscopy to perform oviductal ligation in horses to prepare them for gamete intra-Fallopian transfer transfer [96]. A hand-assisted technique was used to remove a uterine leiomyoma in a standing mare [97]. Laparoscopy has also been used to assist in reproductive therapies. Prostaglandin gel has been applied to the oviduct in infertile mares to successfully reestablish patency [98, 99]. Another report evaluated laparoscopic techniques for investigating the equine oviduct [100]. Brink et al. [101] reported on the use of laparoscopy for raising the uterus in multiparous mares that were not rebreeding. In 5 mares, the broad ligament was “shortened” by use of laparoscopic suturing. All five mares were successfully operated, and 3 of 5 became pregnant in the same breeding season without any further interventions. 

### 17.4. Surgery of the Renal System

 Laparoscopy has been used in bladder surgery of the horse, primarily in the repair of the ruptured bladder [102–104] and for removal of uroliths [93, 105, 106]. In the report by Edwards et al. [102] a stapling device was used to repair the bladder tear in a foal. The foal eventually formed a urolith that required a second surgery. The authors suggested that nonabsorbable staples should not be used, especially if they penetrate the lumen of the bladder. It is generally recommended that only a rapidly absorbing suture material be used in the bladder, and that the mucosa not be penetrated if possible. Laparoscopy has also been described in the resection of umbilical structures [107].

Laparoscopic surgery of the kidneys has been reported [108–111]. While surgery of the kidneys is not common in the horse, a hand assisted laparoscopic approach has allowed the surgeon to perform more procedures with less morbidity than the standard open approach.

### 17.5. Surgery of the Gastrointestinal Tract

 All surgical interventions of the abdomen should include a general exploratory of the peritoneal cavity. A thorough understanding of the in-situ anatomy is a must and has been previously covered. In general a standing position is preferred when the area of interest is in the dorsal abdomen, and a dorsally recumbent position is preferred when the area of interest is in the ventral abdomen. 

When performing standing exploratory surgery, it is always a good idea to prepare both flanks for surgery. It is very helpful to have at least one 45 cm instrument to be able to lift the small colon to evaluate the other side of the abdomen. It is also possible to have someone lift the colon by placing his or her arm in the colon as if performing transrectal palpation. Common structures visible in the standing horse from the left flank include: stomach, diaphragm, spleen, kidney (retroperitoneal), small colon, mesocolon, small intestine, left ovary in mares, left uterine horn in mares, left vaginal ring in males, bladder, and whatever part of the colon that is dorsal. From the right flank the following structures are generally visible; duodenum, epiploic foramen, ventral band of the cecum, small colon, mesocolon, small intestine, right ovary in mares, right uterine horn in mares, right vaginal ring in males, bladder, and whatever part of the colon that is dorsal. It is possible to break down adhesions while performing exploratory surgery. The use of a Ligasure wand will make breaking down adhesions much easier, and results in less hemorrhage than sharp dissection. A subperitoneal cyst was diagnosed with laparoscopy in a Friesian mare, [112] and more recently for work up of inflammatory bowel disease [113].

 The intestinal tract can be evaluated easily in foals in dorsal recumbency, [58, 114] and less easily in adult horses in either the standing or recumbent positions [115]. Laparoscopy can be valuable in the evaluation of horses with abdominal disease or colic [116, 117]. It is important to realize that horses with significant amounts of intestinal distention pose a risk of bowel trauma when inserting the trochars. The small bowel is easier to manipulate than the large bowel. Care should be taken to minimize tears in the bowel during manipulation. Laparoscopy has also been used to evaluate the health of the descending colon after rectal prolapse and tearing of the mesocolon during parturition [118]. Laparoscopy has also been used to diagnose an adenocarcinoma of the small intestine [119]. Laparoscopy has also been used to repair a small intestinal mesenteric rent in a broodmare post-foaling [120]. Two retrospective studies on surgical treatment of colic in miniature horses showed that laparoscopy could be valuable to identify adhesions [121, 122].

 Biopsy of the intestinal tract has been reported [115, 123]. In the study by Bracamonte [123] they compared double layer hand-sewn biopsy techniques with an endoscopic linear stapler biopsy for bursting strength, bursting wall tension, and luminal diameter reduction. They identified that bursting strength and bursting wall tension were significantly lower in the stapling group when compared to the hand-sewn group. The hand-sewn group significantly reduced the luminal size when compared to the stapled group. All biopsy specimens were acceptable for histology, and there were no complications noted.

 There are few reports of endoscopic organ biopsy in the horse. Laparoscopy has been used for liver biopsy [124] and kidney [125]. The main benefits of laparoscopic organ biopsy are that a larger sample can be obtained, and the sample can be taken from an area that shows superficial abnormalities. The biopsy site can also be observed for bleeding after the biopsy has been performed.

 Experimental laparoscopic colopexy was first described in 1998 [126]. The procedure was successfully used in a clinical case 5 years later [127]. The main benefit of laparoscopic colopexy over the traditional open approach is the lack of a long ventral midline surgical approach and the attending healing time.

 The ablation of the nephrosplenic space has become commonplace in equine laparoscopic surgery [128–131]. It has been shown that horses with a left dorsal displacement of the colon over the nephrosplenic space are more likely to reentrap after correction of the displacement. In one report of 44 horses with laparoscopic surgery and an evaluation of 4,852 horses treated for colic over 16 years, there was an incidence of 6% for colonic displacement, and recurrence of 21% in this group of horses [130]. Consequently a technique was developed to close the space using minimally invasive surgical techniques [128]. Laparoscopy has also been used to evaluate a subcapsular splenic hematoma in a horse [132].

 Laparoscopic adhesiolysis has been studied in the horse [114, 133–135]. The clinical case report described a 14-year-old Arabian mare with adhesions of the small intestine to the paramedian incision after surgical complications following a paramedian ovariectomy. The adhesions were broken down laparoscopically and the mare recovered [133]. The experimental studies showed that radio surgery was successful in reducing peritoneal adhesions, but that addition of 0.5% ferric hyaluronate gel would reduce the likelihood of future adhesion formation [135]. Abscesses have also been evaluated and treated with the use of laparoscopic surgery [136].

 Iatrogenic tears of the descending and terminal colon have always been challenging for the equine surgeon to repair. A report of experimentally induced rectal tears highlighted the potential benefit of minimally invasive surgery [137]. In this study, horses had iatrogenic rectal lacerations created which were successfully repaired via laparoscopy.

### 17.6. New Techniques

 Recent advances in human laparoscopy are being evaluated in equine laparoscopy. Most notably is the evaluation of natural orifice transluminal endoscopic surgery (NOTES) [138]. In this study, it was determined that abdominal exploration was adequate through either the left or right transvaginal approach. Structures that could be evaluated were the left kidney, spleen, nephrosplenic space, stomach, cecum, duodenum, left and right ovaries, diaphragm, caudal peritoneal reflection, and inconsistently the liver. Caron and Mehler [139] described the use of mesh for repair of umbilical hernias in five horses. The mesh is placed against the body wall, but not retroperitoneally. The results were considered good for function and variable cosmetically.

## 18. Complications

 Complications associated with laparoscopic surgery have been well documented [20, 21, 52, 140, 141]. The challenges involved in laparoscopic surgery lead to most of the complications associated with laparoscopic surgery. These challenges include working on a two-dimensional monitor that limits depth perception, the fulcrum of the body wall, placing trochars through a small skin incision without being able to observe what lies in their path, limited mobility within the peritoneal space, and lack of training. The best technique to treat complications is to reduce the occurrence of complications [142].
